# Adaptive Evolution of Eel Fluorescent Proteins from Fatty Acid Binding Proteins Produces Bright Fluorescence in the Marine Environment

**DOI:** 10.1371/journal.pone.0140972

**Published:** 2015-11-11

**Authors:** David F. Gruber, Jean P. Gaffney, Shaadi Mehr, Rob DeSalle, John S. Sparks, Jelena Platisa, Vincent A. Pieribone

**Affiliations:** 1 Baruch College, Department of Natural Sciences, City University of New York, New York, New York, United States of America; 2 The Graduate Center, Program in Biology, City University of New York, New York, New York, United States of America; 3 State University of New York, Biological Science Department, College at Old Westbury, Old Westbury, New York, United States of America; 4 American Museum of Natural History, Sackler Institute for Comparative Genomics,Central Park W at 79th St, New York, New York, United States of America; 5 American Museum of Natural History, Department of Ichthyology, Division of Vertebrate Zoology, American Museum of Natural History, New York, New York, United States of America; 6 The John B. Pierce Laboratory, Inc., New Haven, Connecticut, United States of America; 7 Cellular and Molecular Physiology, Yale University School of Medicine, New Haven, Connecticut, United States of America; 8 Department of Neurobiology, Yale University School of Medicine, New Haven, Connecticut, United States of America; Universidade Federal do Rio Grande do Sul, BRAZIL

## Abstract

We report the identification and characterization of two new members of a family of bilirubin-inducible fluorescent proteins (FPs) from marine chlopsid eels and demonstrate a key region of the sequence that serves as an evolutionary switch from non-fluorescent to fluorescent fatty acid-binding proteins (FABPs). Using transcriptomic analysis of two species of brightly fluorescent *Kaupichthys* eels (*Kaupichthys hyoproroides and Kaupichthys n*. *sp*.*)*, two new FPs were identified, cloned and characterized (Chlopsid FP I and Chlopsid FP II). We then performed phylogenetic analysis on 210 FABPs, spanning 16 vertebrate orders, and including 163 vertebrate taxa. We show that the fluorescent FPs diverged as a protein family and are the sister group to brain FABPs. Our results indicate that the evolution of this family involved at least three gene duplication events. We show that fluorescent FABPs possess a unique, conserved tripeptide Gly-Pro-Pro sequence motif, which is not found in non-fluorescent fatty acid binding proteins. This motif arose from a duplication event of the FABP brain isoforms and was under strong purifying selection, leading to the classification of this new FP family. Residues adjacent to the motif are under strong positive selection, suggesting a further refinement of the eel protein’s fluorescent properties. We present a phylogenetic reconstruction of this emerging FP family and describe additional fluorescent FABP members from groups of distantly related eels. The elucidation of this class of fish FPs with diverse properties provides new templates for the development of protein-based fluorescent tools. The evolutionary adaptation from fatty acid-binding proteins to fluorescent fatty acid-binding proteins raises intrigue as to the functional role of bright green fluorescence in this cryptic genus of reclusive eels that inhabit a blue, nearly monochromatic, marine environment.

## Introduction

The photic marine environment is proving to be a crucible of evolution for novel biofluorescent molecules. With increasing depth in the ocean, the intensity of sunlight decreases in an approximately exponential manner and the spectral quality of light also changes, becoming increasingly restricted to a narrow range of wavelengths of blue light (470–490nm)[1]. This was first qualitatively described by Beebe [2] whose firsthand account conveys how the red, orange, yellow, and green components of sunlight disappeared during his bathysphere descent into the mesopelagic zone, leaving a predominately blue environment. Marine organisms biofluoresce by absorbing the dominant blue light, and reemitting it at a longer, lower energy wavelength, visually resulting in green, orange, and red fluorescence. Following the seminal discovery of green fluorescent protein (GFP) from a hydrozoan jellyfish in 1962 [3], fluorescent proteins (FPs) have been found in numerous anthozoans, primarily scleractinian corals [4,5], copepods [6], amphioxus [7,8], ctenophores [9] and most recently, fishes [10,11].

Biofluorescence has been most extensively studied in scleractinian corals, where it has been hypothesized to function in photoprotection [12], antioxidation [13], regulation of symbiotic dinoflagellates [14], visual contrast [5], coral health [15] and photoacclimation [16]. The finding that biofluorescence is phylogenetically widespread and phenotypically variable in marine fishes [17] highlights many interesting new questions as to the role of biofluorescence in groups with advanced visual capability. Many fishes have been shown to possess yellow intraocular (lenses or cornea) filters [18], which could potentially function as long-pass filters and could enable enhanced perception of biofluorescence.

**Fig 1:**
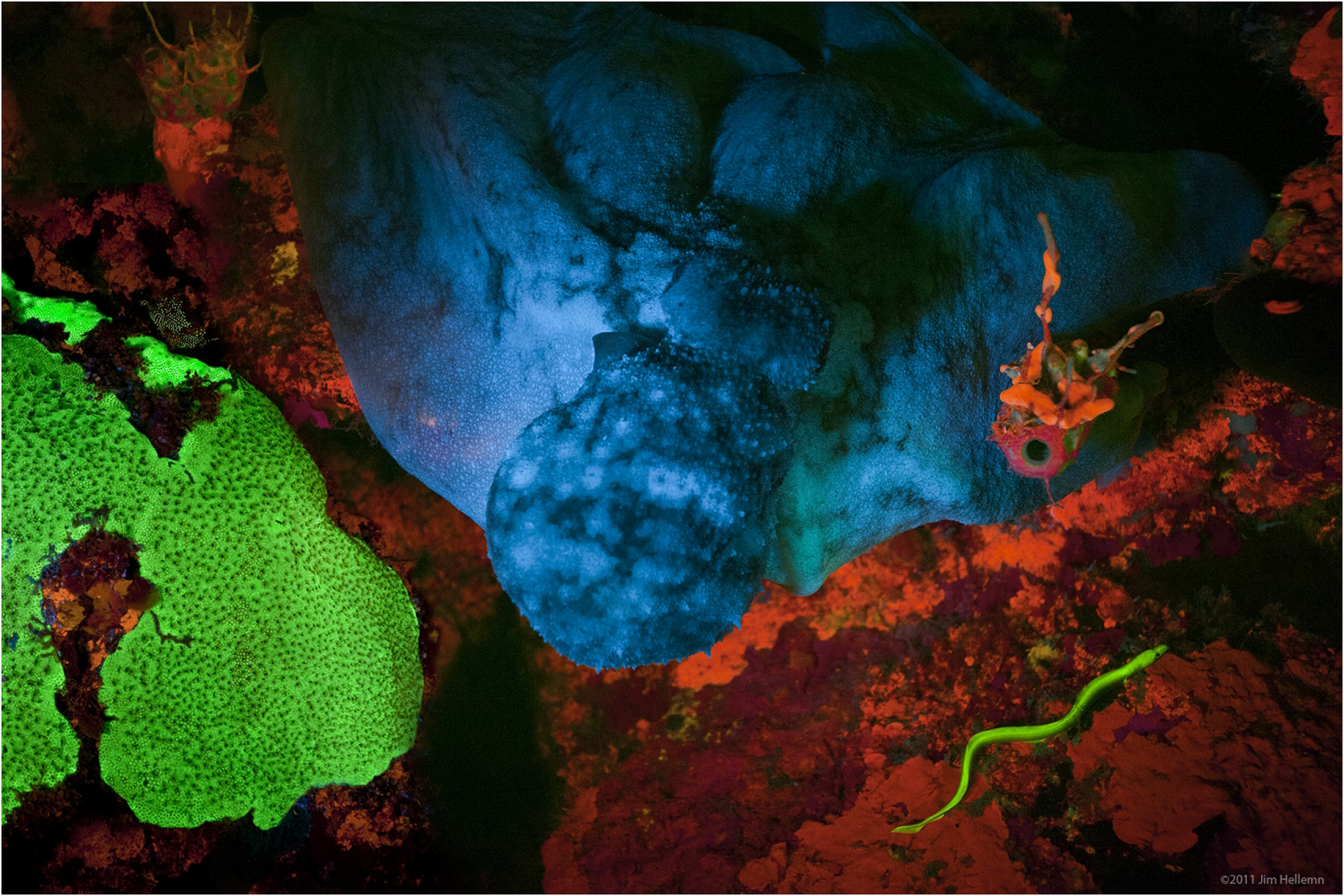
Biofluorescent Chlopsidae (lower right) photographed in Little Cayman Island.

**Fig 2:**
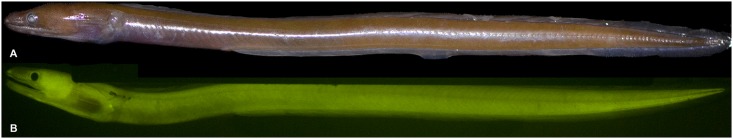
*Kaupichthys hyoproroides* collected in Bahamas and used in this study. A) White light; B) Green fluorescence.

**Fig 3:**
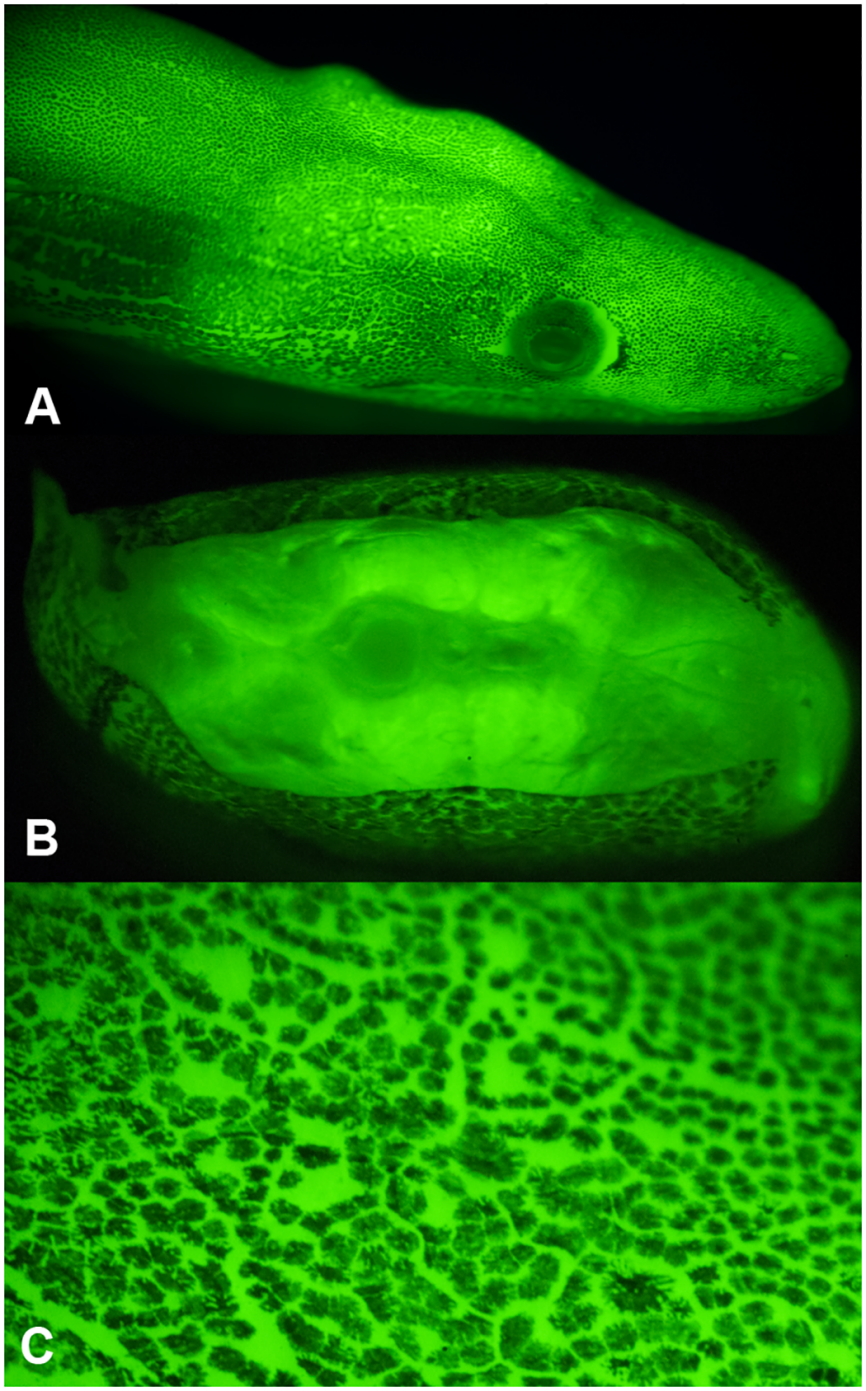
Details of green fluorescence of *Kaupichthys sp*. A) Head fluorescence, B) Cross-section through demonstrating fluorescence in the musculature. C) Close up of skin revealing dark pigmented regions interspersed with fluorescence arising from both the skin and internal musculature.

We serendipitously imaged an intensely green fluorescent false moray (family Chlopsidae) eel while studying biofluorescent coral during a 2011 expedition to Little Cayman Island in the Caribbean Sea (Fig 1). To our knowledge, this marked the first time that a brightly green fluorescent vertebrate was imaged in its natural habitat. Its fluorescence matched the intensity of adjacent brightly fluorescent corals. This discovery led to a series of expeditions to the Caribbean and South Pacific where several species of biofluorescent eels, representing several anguilliform families, were collected and analyzed [17]. In this study, we report the discovery and characterization of novel eel fluorescent proteins (Chlopsid FP I from *Kaupichthys hyoproroides* and Chlopsid FP II from *Kaupichthys* n. sp.) based on transcriptome analysis of the false moray eels, *Kaupichthys hyoproroides* (Fig 2) and *Kaupichthys* n. sp., the later representing a heretofore undescribed species (Fig 3).

Previously, a bilirubin inducible fluorescent protein, UnaG, was identified from *Anguilla japonica*, a species of eel used extensively in aquaculture [10], and was later fully characterized [11]. Here we identify, clone and characterize two new members of this FP family and demonstrate that fluorescent FABPs have a unique tri-peptide sequence motif (Gly-Pro-Pro) inserted in a loop between two β sheets, which is not found in other non-fluorescent FABPs. Chlopsid FP I and Chlopsid FP II exhibit blue shifted emission spectra when compared to UnaG. We show using analysis of dN/dS skew (MEME option in Datamonkey) [19] that this sequence motif arose from a duplication event of the FABP brain isoforms and was under strong purifying selection during the evolution of the family leading to this new florescent protein family. In addition, residues adjacent to the motif are shown to be under strong positive selection, which we suggest is a further refinement of the fluorescent properties of the proteins in eels. Here we expand on the identification, biochemical characterization, and phylogentic grouping of this new family of fluorescent eel proteins as first identified by Kumagai et al. [11].

## Material and Methods

### Fluorescent eel collection and identification

Research, collecting and export permits were obtained from the government of the Bahamas, from the Ministry of Fisheries and Ministry of Environment, Honiara, Solomon Islands, and from the Department of Environment, Cayman Islands. This study was approved and carried out in strict accordance with the recommendations in the Guidelines for the Use of Fishes in Research of the American Fisheries Society and the American Museum of Natural History's Institutional Animal Care and Use Committee (IACUC). Fishes were collected via SCUBA, using both standard open circuit systems and closed circuit rebreathers, via the application of rotenone and quinaldine to a targeted variety of shallow water to deep (mesophotic) habitats in each sampling location where collecting was permitted.

### Fluorescent Macro Photography

Both of the chlopsid eel specimens utilized for the transcriptome and protein work described herein (*Kaupichthys hyoproroides* and *Kaupichthys* n. sp.) were immediately placed on ice to preserve coloration and digitally imaged upon return to shore. Prior to imaging, the specimens were subsequently scanned for fluorescence using bright LED light sources equipped with excitation filters and observed using emission filter glasses/goggles. *Kaupichthys hyoproroides* and *Kaupichthys* n. sp. were placed in a narrow photographic tank and held against a thin plate glass front. Fluorescent macro images [4928 x 3264 (Nikon D7000); 2180 x 1800 pixel (Nikon D300S)] were produced in a dark room by covering the flash (Nikon SB 600 and SB 800) with band-pass (BP) excitation filters (Omega Optical, Brattleboro, VT) and attaching long-pass (LP) (Semrock, Rochester, NY) filters to the front of the camera lens. Two different excitation/emission filter pairs were tested on each sample to stimulate the strongest fluorescence emission: excitation 450–500 nm, emission 514 LP; excitation 500–550 nm, emission 555 and 561 LP. All images were obtained within two hours of collection and the sample was immediately frozen in a liquid nitrogen dry shipper for transport. Cross-sectional images of specimens were generated using a Zeiss-Axio Zoom V16 stereo fluorescent microscope affixed with a Nikon D4 camera (Fig 3).

### Fluorescent Protein Isolation from *Kaupichthys* Tissue

**Fig 4:**
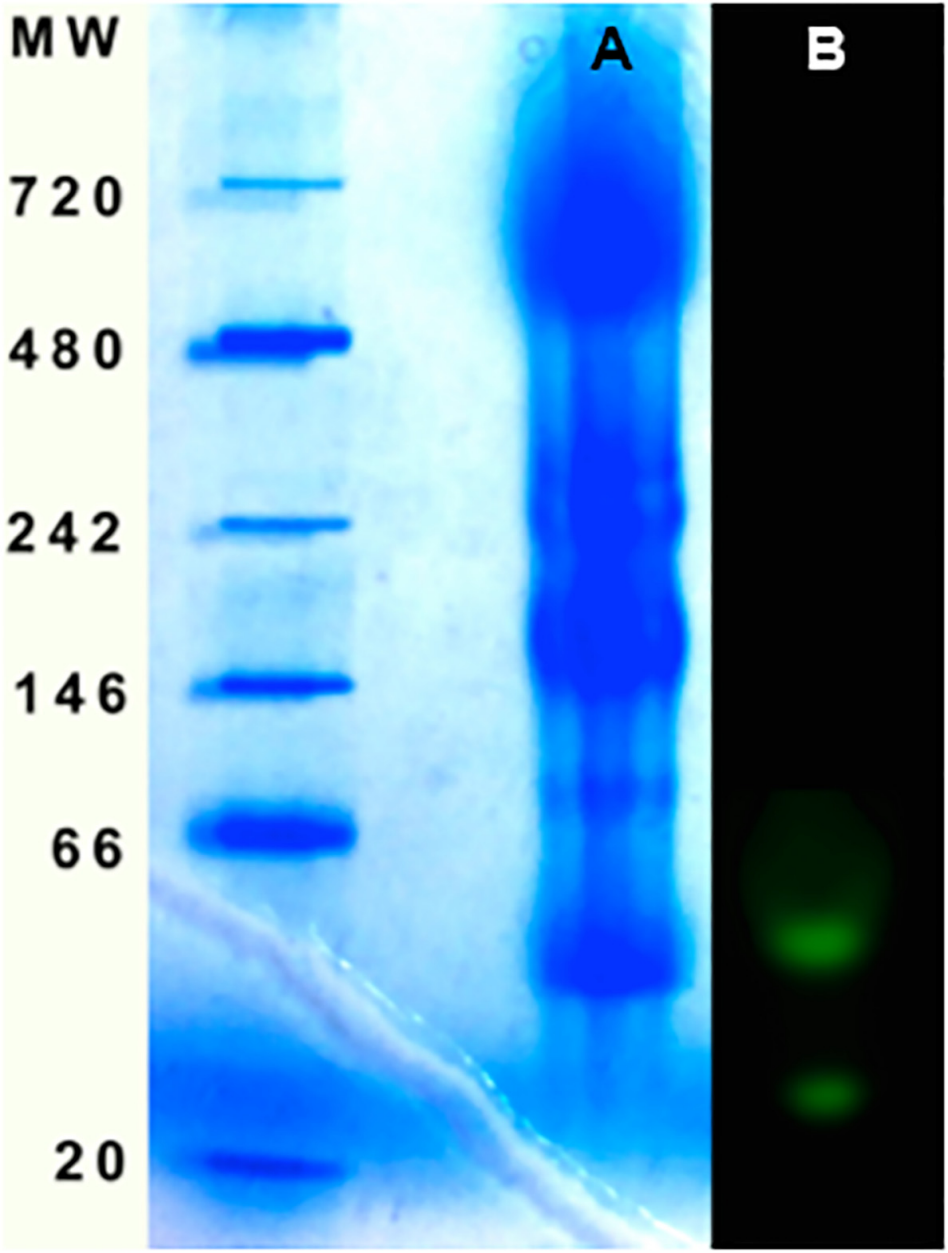
Native gel of tissue homogenate from *Kaupichthys hyoproroides*. A) Coomassie stained gel; B) Fluorescent bands imaged under illumination with blue light.

A native protein extract was prepared from a small cross-section of eel musculature and was run on a non-denaturing PAGE gel stained with Coomassie Brilliant Blue. Using fluorescent imaging, two bands were observed that exhibited strong green fluorescence (Fig 4).

### RNA Extraction and Transcriptome Sequencing

Total RNA was extracted from the caudal musculature of two distinct species of chlopsid eel *K*. *hyoproroides* and an undescribed species referred to as *Kaupichthys* n. sp. Muscle tissue was homogenized in TriZol reagent (Life Technologies, Carlsbad, CA), and the total RNA was precipitated with isopropanol and dissolved in ddH_2_O. The quality of RNA was assessed on a 2100 Bioanalyzer and with agarose gel electrophoresis. The total RNA was pooled for library preparation using a Hi-seq RNA sample preparation kit (Illumina Inc, San Diego, CA) according to the manufacturer’s protocol. Sequencing was performed in a multiplexed lane of a flow cell using Illumina Hi-seq 2000. FASTQ file generation was performed by CASAVA ver. 1.8.2 (lllumina). Reads were quality checked with FASTQC [20]. Low quality reads and reads containing Illumina adapters were trimmed with Trimmomatic [21]. Reads contaminated with vectors were removed using the NCBI vector database with in-house Perl scripts [22]. Clean reads were uploaded into the NCBI (SRA: SRS493036, Biosample: SAMN02378295).

Trinity [23] was used to generate *de novo* assembled sequences for downstream analyses (Table 1). Cleaned, assembled contigs have been deposited into the NCBI Transcriptome Shotgun Assembly database under the following accession numbers: PRJNA192511 for *Kaupichthys hyoproroides* and accession PRJNA223153 for *Kaupichthys* n. sp.

**Table 1:** Summary statistics for individual assemblies of *Kaupichthys hyoproroides* and *Kaupichthys* n. sp. (Chlopsid I and Chlopsid II, respectively).

Eel	Assembly	Transcripts >200bp	N50 (bp)	Mean contig length (bp)	Max contig length (bp)	Total number of bases
*K*. *hyoproroides*	TransABySS	206,683	877	672	8,547	138,913,931
*K*. *hyoproroides*	Trinity	84,610	880	641	13,309	54,235,411
*Kaupichthys* n. sp.	Trinity	74,448	610	502	7,558	37,356,156

### *In Silico* Quantification of Transcripts

In order to identify the transcript quality, we mapped the reads of *Kaupichthys hyoproroides* back onto the non-redundant set of assembled transcripts using Bowtie [24]. Gene coverage levels were determined using a Perl script to calculate the RPKM [25]. A total of 109,268,961 (76.67%) reads had at least one reported alignment. The minimum coverage of a transcript was 0.03 FPKM and the maximum was 62,622, with an average of 9.44, indicating a wide range of gene expression (Table 2). Contigs with a RPKM smaller than one were removed for downstream analysis. Among these, 65,877 (77.85%) transcripts had a FPKM >1, with an average of 11.93. Also, two transcripts had FPKMs larger than 20,000, with homology to parvalbumin and muscle related actin, with a FPKM of 24,006 and 62,622, respectively. This level of abundance is expected given that these transcripts were generated from muscle tissue. The EMBOSS package [26] was used to generate all possible open reading frames (ORFs) from stop to stop for each assembled contig.

**Table 2:** Summary statistics of read counts and coverage of *Kaupichthys hyoproroides*.

Total number of reads	142,526,414
Number of read used reads for assembly	109,268,961 (76.67%)
Number of unused reads	33,257,453 (23.33%)
Number of non-redundant transcripts (>200 bp)	84,610
Number of transcripts with coverage fpkm >1	65,877
Average coverage for contigs with coverage fpkm >1	11.93
Average number of reads mapped per contigs	1,649.71

### Protein Search

**Fig 5:**
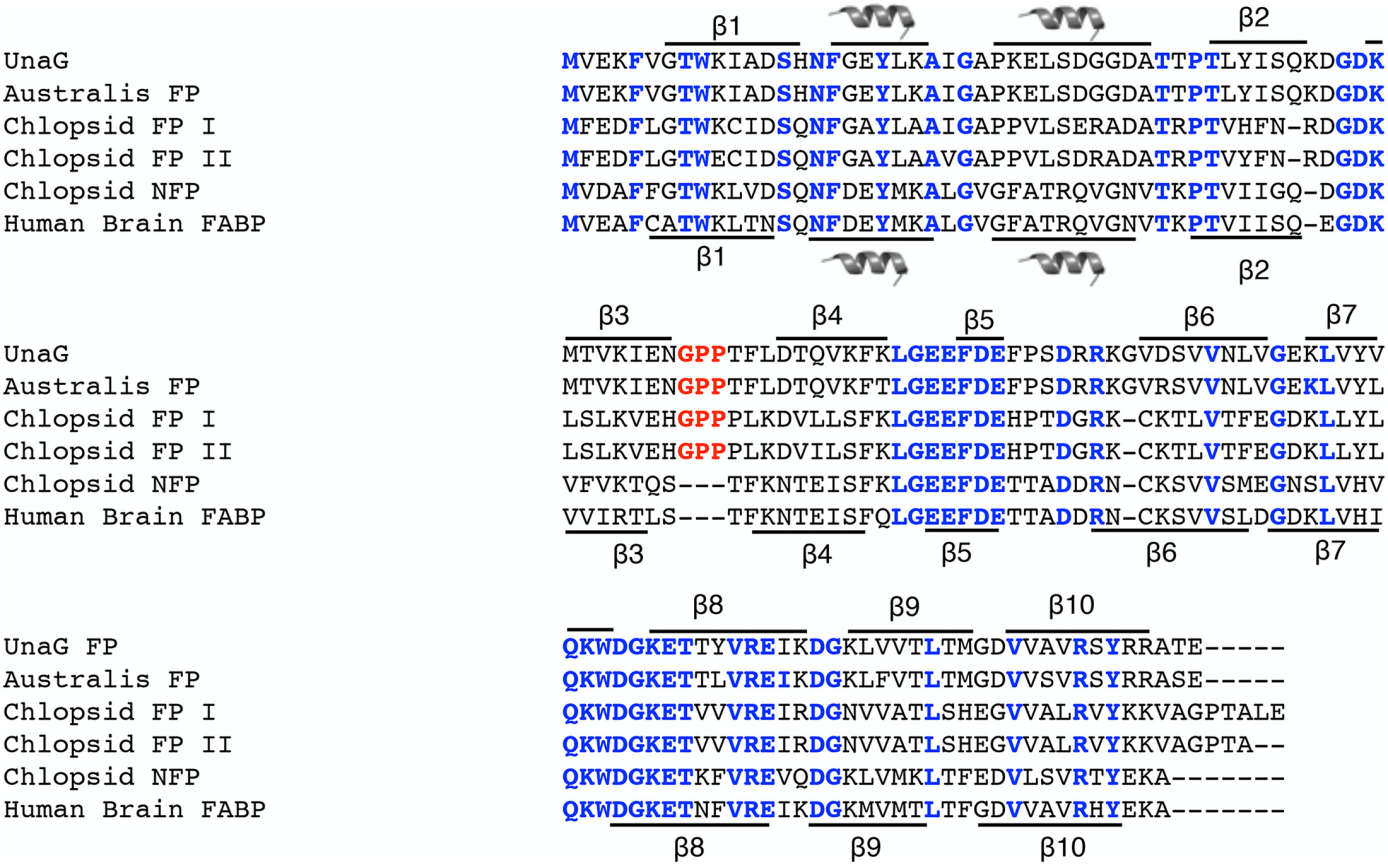
Sequence alignment of fluorescent FABPs from eels with a non-fluorescent FABP from *Kaupichthys hyoproroides* (Chlopsid NFP) and human brain FABP-7. Residues highlighted in blue show areas of homology. The GPP sequence motif is highlighted in red.

The EMBOSS package [26] was used to generate all possible open reading frames (ORFs) from stop to stop for each assembled contig. ORF sequences were searched for FABP using BlastP [27]. Target ORFs with an unusual sequence motif (Gly-Pro-Pro motif) on a loop between two beta sheets in the FABP sequence were selected as potential fluorescent sequences. An alignment of the FPs is shown in Fig 5.

### Phylogenetic Analysis

#### Sequences and Tree Construction

The phylogenetic matrix we eventually used has 210 terminals in it. These terminals were obtained using a BLAST search with human FABP7 with an e-value cutoff of e-25. Once we determined that there were two FABP isomers in the majority of the vertebrate taxa with hits at e-25 (one heart isomer and one liver), we then searched the database further for the two paralogs for all of the taxa. This final search resulted in a matrix of 210 terminals over 163 vertebrate species (See S1 Table for accession numbers and abbreviations used in the matrix and phylogenetic trees). The DNA sequences were aligned using TranslatorX [28], which provides both DNA and amino acid sequence alignments that agree at all codons. The DNA sequences were translated into amino acid sequences and these two data sets were formatted into Nexus (for use in PAUP)[29] and Phylip formats (for use in RaxML Blackbox). The aligned sequences are provided in S2 Table. Maximum likelihood (ML) and maximum parsimony (MP) trees were generated using RaxML blackbox [30] and PAUP. Bootstrap trees for the two methods were also generated. We used Modeltest [31] to determine the best model for the DNA sequence matrix and ProTest [32] for the amino acid sequence matrix. The GAMMA+P-Invar model was used for DNA sequence ML analysis. The WAG with GAMMA+P-Invar model was used for ML analysis of proteins. Bayesian analysis (BY) use 1,000,000 generations with the GAMMA+P-Invar model with default priors (convergence of chains was obtained with this number of generations). In all, ten trees were generated and compared for congruence, where the data source [DNA or Protein] is listed first followed by a slash, then the phylogenetic criterion [MP, ML or BY] listed second followed by a slash, and finally the robustness criterion used [bootstrap or single best tree]). 1) DNA/MP/best tree, 2) DNA/MP/bootstrap, 3) Protein/MP/best tree, 4) DNA/MP/bootstrap (S1 Fig); 5) DNA/ML/best tree, 6) DNA/ML/bootstrap, 7) Protein/ML/best tree, 8) Protein/ML/bootstrap (S2 Fig); 9) Protein/BY and 10) DNA/BY (S3 Fig).

#### Analysis of dN/dS ratios

For dN/dS skew detection at the residue level we used the MEME option in Datamonkey (http://www.datamonkey.org/) and for examining dN/dS skew on branches of the phylogeny (BREL) for these proteins we used HYPHY (http://hyphy.org/w/index.php/Main_Page). The MEME option can use either a NJ tree or a user supplied tree. Hence we used the MEME option with two input trees—the NJ tree and the ML tree obtained by phylogenetic analysis of the data. A model of sequence evolution is also required for MEME and so we used the default model as supplied in Datamonkey and the optimal model as derived from the “automatic model selection tool” option in Datamonkey. The optimal model from Datamonkey automatic model selection tool was 012032. This approach required four separate MEME runs that resulted in from six to eight sites under positive Darwinian selection depending on the parameters of the analysis (raw results presented in S3 Table). The dN/dS branch analysis (BREL) was accomplished with the HYPHY program using the ML tree as an input tree and computing statistics only for internal branches. We also used the PRIME option in Datamonkey to characterize the potential change in properties of the residues that experience positive dN/dS skew. PRIME is a variation of MEME that detects residue changes that can be categorized as changes in the original property of the amino acid. There are five categorical changes that can be detected using prime—polarity index, secondary structure factor, volume, refractivity/heat capacity and charge/iso-electric point [33].

### Protein Expression and Purification

Candidate ORFs were selected from eel transcriptome data containing an unusual sequence motif (Gly-Pro-Pro) on a loop between two beta sheets in the FABP sequence. The genes for Chlopsid FP I (*Kaupichthys hyoproroides*) and Chlopsid FP II (*Kaupichthys* n. sp.) were synthesized (GenScript USA) and cloned into the NdeI-ZhoI cloning site of a pET-24b(+) vector utilizing the C-terminal His-Tag. Recombinant protein was expressed in a soluble form in BL21(DE3) *E*. *coli* cells and purified using Ni-affinity chromatography on an AKTA-Prime FPLC, eluting with 50 mM Tris and 300 mM imidazole, pH 8.0. The protein was dialyzed against 50 mM Tris and 20 mM NaCl to remove imidazole, and was concentrated using an Amicon Ultra centrifugal concentrator (m.w.c.o. 3000). Protein purity was confirmed using SDS-PAGE. Protein concentration was determined by A_280_ measurements, using calculated extinction coefficients of 15,300 M^-1^cm^-1^ for Chlopsid FP I, and 16,600 M^-1^cm^-1^ for Chlopsid FP II. Bilirubin (Sigma-Aldrich, USA) was dissolved in 0.1 M NaOH and immediately diluted in 50 mM Tris buffer, pH 8.0, for use in experiments.

### Fluorescence Spectroscopy

Fluorescence excitation and emission spectra were recorded using a F-7000 Hitachi Fluorescence Spectrometer.

### Expression of Chlopsid FP I in HEK293 Cells

A pCS2+ vector was used for cloning of Chlopsid FP I for expression in HEK293 cells (ATCC, USA) using Kpn-SphI cloning sites. Plasmids were prepared by Genscript USA, Inc. The expression was driven in HEK293 cells (ATCC, USA) by CMV promotor. The HEK293 cell line was maintained in Dulbecco’s Modified Eagle Medium (High Glucose) (DMEM) (Invitrogen, USA), supplemented with 10% fetal bovine serum (FBS) (Sigma-Aldrich, USA), in a 37°C incubator with 5% CO_2_. Transient transfection was performed using 2 μg of DNA per 35 mm dish and 5μg of Lipofectamine 2000 (Invitrogen, NY). Cells were imaged on a custom-made 2-photon microscope using a Chameleon Ti-Sapphire laser (Coherent Inc, CA) and water immersion 20x/0.95 N.A. objective (Olympus, Japan). Images were taken using 850nm laser light with power of 15mW at the objective.

## Results

During a January 2011 fluorescent coral reef photomosaic-imaging trip to Bloody Bay Wall off Little Cayman Island in the Caribbean, a green fluorescent chlopsid eel, likely belonging to the genus *Kaupichthys*, was serendipitously photographed (Fig 1). This finding was presented in the American Museum of Natural History exhibit, “*Creatures of Light*: *Nature’s Bioluminescence*” in 2012. The animal seen in the photograph was identified as belonging to the Chlopsidae family of eels, one the most poorly known families of the order Anguilliformes. Chlopsids exhibit extremely cryptic behavior and are rarely seen alive in their natural habitat [34]. Most existing specimens were obtained using piscicides (e.g., rotenone).

Surprised by this animal’s bright, visible green fluorescence, we embarked upon a collection expedition to Lee Stocking Island in the Bahamas where we ultimately collected single specimens of two brightly biofluorescent chlopid eel species, *Kaupichthys hyoproroides* and *Kaupichthys* n. sp. (Figs 2 and 3). *Kaupichthys hyoproroides* reaches a maximum length of about 250 mm and spends most of its life hiding in holes or crevices of coral reef areas or sea grass beds [34]. In cross-section, the fluorescence was found to be bright throughout the muscle tissue and also within the skin in specimens of both species. Muscle tissue was dissected from both species from which we isolated mRNA as well as a highly fluorescent soluble protein extract. The mRNA was used for HiSeq transciptomic analysis.

We subjected muscle tissue extract from *K*. *hyoproroides* to NativeBlue (Invitrogen) non-denaturing gel electrophoresis. Under blue light and imaged with a yellow filter, two bands were observed that exhibited strong green fluorescence (Fig 4). In addition, upon boiling the extract, the fluorescence disappeared. These findings led us to conclude that the fluorescence was most likely arising from a protein. However, transcriptome analysis of the muscle mRNA failed to produce any GFP-like sequences and the fluorescence emission spectrum of the protein extract (not shown) differed from eGFP.

**Fig 6:**
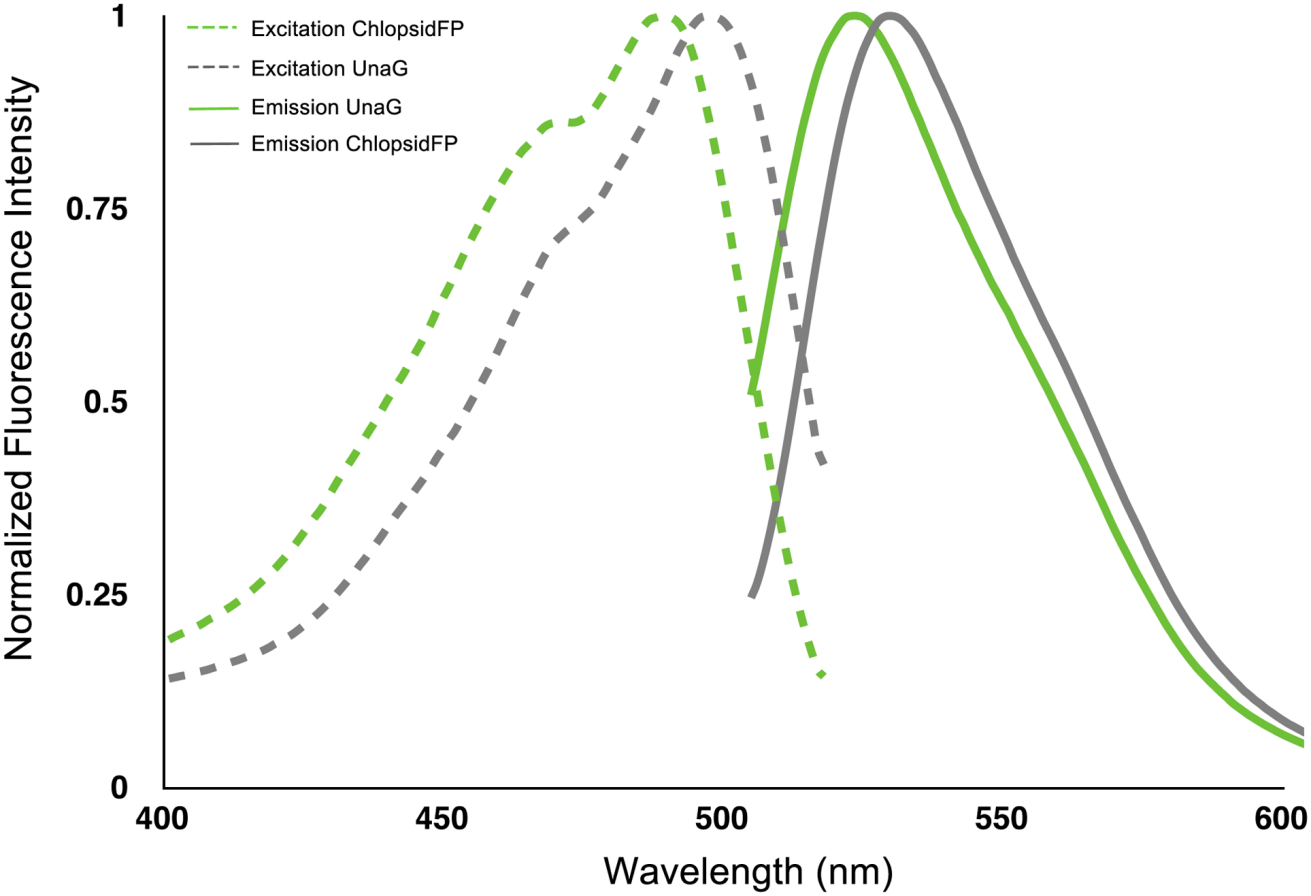
Excitation/emission spectra of Chlopsid FP I and UnaG.

Hayashi and Toda reported that *Anquilla japonica* (heavily farmed in Japanese aquaculture and a historical staple of Japanese cuisine) was weakly green fluorescent [10]. They purified a fluorescent protein from *A*. *japonica* muscle tissue and isolated and sequenced several peptide fragments. Some of the peptides isolated were found to be homologous to previously published fish fatty acid binding proteins (FABPs). Based on these results, we performed a crude purification of the fluorescent bands from the electrophoresis of eel muscle protein extract and subjected it to mass spectroscopy. We identified the full-length sequences of these proteins in the *Kaupichthys* transcriptome data and synthesized two genes exhibiting the highest homology to the proteins identified by Hayashi and Toda [10]. However, expression of these proteins did not produce visible fluorescence in either *E*. *coli* or mammalian cells. Then in 2013, Kumagai et al., published a full characterization of the fluorescent protein from *A*. *japonica* [11]. The protein, termed UnaG, is a novel member of the FABP family, and the fluorophore was found to be a bound bilirubin molecule. Unlike the non-fluorescent FAP sequences we had synthesized from *Kaupichthys*, we noticed that UnaG had an insertion of the tri-peptide Gly-Pro-Pro. We then re-examined the *Kaupichthys hyoproroides* and *Kaupichthys* n. sp. data and found a single transcript in each of the two species’ transcriptomes that encoded an FABP including the Gly-Pro-Pro insertion. We synthesized the proteins containing this motif and both showed strong green fluorescence in mammalian cells and E. coli upon addition of exogenous bilirubin. These proteins, termed Chlopsid FP I and Chlopsid FP II, are orthologs of UnaG (Fig 5) and have ex/em spectra of 489nm/523nm (Fig 6).

### Phylogenetic Analysis

**Fig 7:**
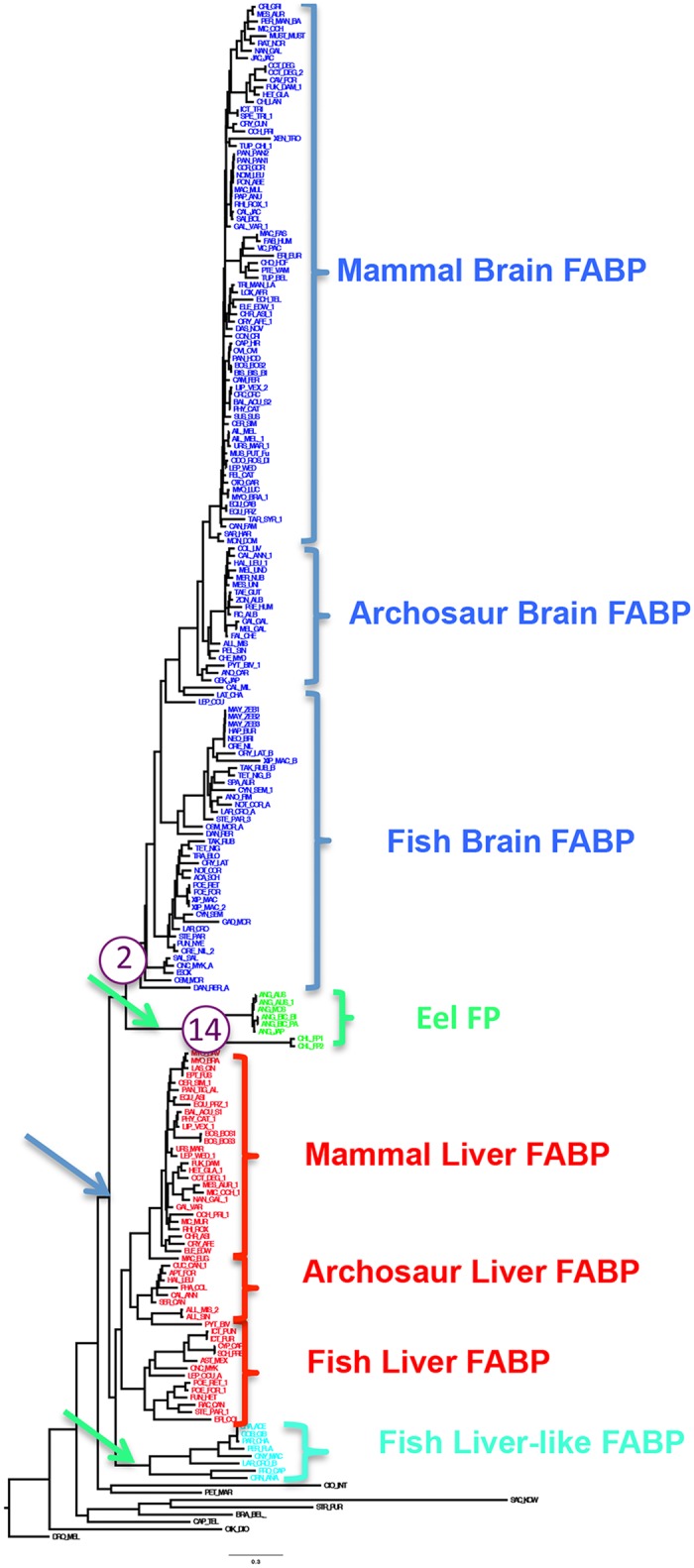
Phylogenetic tree generated by Maximum likelihood analysis in RaxML Blackbox. See text for details of analysis. The arrows in the tree indicate potential branches where duplications occur to explain the paralog patterns in the gene family. The purple circles indicate the two branches where significant dN/dS skew occurs, and the number inside of the circle refers to the magnitude of the dN/dS skew.

The phylogenetic patterns we observed (Fig 7; S1, S2 and S3 Figs) indicate that the FABP gene family was generated by at least two duplication events. These duplications possibly coincide with the 1R and 2R duplications in the common ancestor of vertebrates [35]. Alternatively it is possible that a single duplication gave rise to the two major kinds of FABPs and independent duplications in specific fish lineages led to the eel FPs and the Fish Liver-like FABPs. While there is some variation as to the placement of the eel FPs in relation to the liver and brain FABPs depending on the analysis parameters and optimality criteria, the DNA ML analysis and the Bayesian and MP trees place the Eel FPs either sister to or within the brain FABPs. Our phylogenetic analyses therefore suggest that the eel FPs are more closely related to the brain FABPs than to the liver FABP proteins (Fig 7).

To examine if the neofunctionalization of the FPs as a result of duplications involved sequence specific changes or dN/dS skew, and potentially natural selection, we examined which branches are evolving under different dN/dS skew. The results of this analysis are shown in Fig 7. Two nodes showed statistically significant difference in skew. The node leading to the brain FABP7 in mammals and birds has a dN/dS ratio of > 2.0. The branch leading to the FPs (both *Kaupichthys* and *Anguilla* FPs) has a dN/dS skew >14, indicating strong sequence change in the common ancestor of these eel FPs, similar to what has been reported for opsins [36,37].

**Fig 8:**
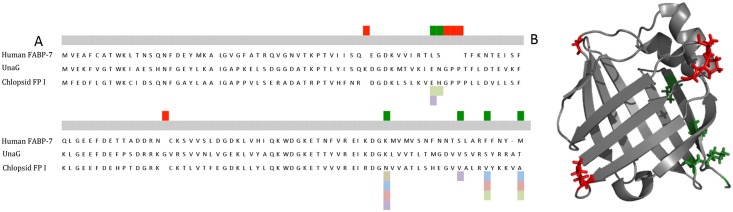
Diagram showing the alignment of FABP’s and structure of UnaG. A) Alignment of human brain FABP-7, UnaG and Chlopsid FP I. Red rectangles show residues that are inserted in the eel FP. Green rectangles show residues under dN/dS skew. Grey rectangles indicate polarity index. Blue highlights secondary structure factors. Pink highlights volume. Light green highlights refractivity/heat capacity and purple highlights charge/iso-electric point. When multiple colors appear at a site, this means that more than a single method detected changes or skew at those positions. B) Crystal structure of UnaG (pdb 4I3B) showing areas that are inserted in the eel FP as well as green showing residues under dN/dS skew.

A site-by-site analysis of dN/dS skew in the FABP7 family of proteins indicates several sites in the protein that show significant skew using the MEME option in Datamonkey [19] under different phylogenetic hypotheses and models of evolution. The number of sites under positive dN/dS skew range from eight (for the NJ tree with the best model) to six (for both of the ML tree analysis regardless of model). We will discuss the more conservative results for the ML tree here, but it should be noted that there is broad overlap in the inferences made regardless of tree or model of sequence evolution. The Gly-Pro-Pro motif shows strong purifying selection as it is a conserved motif (dN = 0; dN/dS = 0) in all organisms where it is found. Fig 8 maps the location of the inserted Gly-Pro-Pro residues in residue positions 59, 60 and 61 of the eel FPs. The two amino acids preceding the conserved Gly-Pro-Pro insertion sites in FPs (residue positions 57 and 58) appear to be under strong positive selection. This pattern might suggest that these sites are actively affected by natural selection as a result of the Gly-Pro-Pro insertion in this FP. In addition, there are four sites under positive selection in the carboxy terminus of the protein. When we examine the six sites we observe to have positively skewed dN/dS ratios for altered protein properties we find that the two residues that are adjacent to the Gly-Pro-Pro motif that show dN/dS skew are changing in their refractivity and heat capacity. The other four residues toward the carboxy terminus that show dN/dS skew are changing in their polarity index, their secondary structure factor and in their volume as well as isoelectric point and Refractivity/Heat Capacity.

### Properties of fluorescent FABPs

Chlopsid FP I and Chlopsid FP II exhibit a slightly blue-shifted fluorescence excitation and emission spectra compared to UnaG (498 nm/527nm for UnaG vs 489nm/523nm ex/em for Chlopsid FP I and Chlopsid FP II (Fig 6). Previous work with UnaG showed that mutation of asparagine-57 to an alanine preceding the GPP motif causes quenching of fluorescence [11]. In Chlopsid FP I and Chlopsid FP II this amino acid is a histidine. This difference in amino acid sequence can potentially explain the fluorescence shift, due to an increase in the π conjugation of the system. This change in fluorescence emission spectra demonstrates that it is possible to make changes to the amino acids around the conjugated bilirubin, which can alter the fluorescence spectrum of the protein.

The fluorescence quantum yield of Chlopsid FP I was determined to be 0.47. Chlopsid FP II had a quantum yield of 0.37. These values are close to the reported quantum yield for UnaG of 0.51 [11] (Table 3). Two prolines in the Gly-Pro-Pro sequence motif were mutated to glycine. This mutation resulted in a decreased quantum yield of 0.11.

**Table 3:** Table of properties for fluorescent proteins.

Fluorescent Protein	Ex/Em maxima (nm)	Fluorescence quantum yield	O_2_ requirement	Number of amino acids	Molecular weight (kDa)
Chlopsid FP I	489/523	0.47	no	142	15.8
Chlopsid FP II	489/523	0.37	no	140	15.5
Chlopsid FP I-GGG Mutant	489/523	0.11	no	142	15.3
UnaG [11]	498/527	0.51	no	139	16.5

### Origins and specificity of eel fluorescent FABPs

Chlopsid FP I and Chlopsid FP II are 94% homologous to each other, yet exhibit only 55% sequence homology to UnaG (Fig 5). We sought to determine how unique the Gly-Pro-Pro sequence is amongst the enormous number of FABPs that have been identified across the animal kingdom. The UnaG, Chlopsid FP I and Chlopsid FP II were used as bait for other vertebrate FABPs. FABP DNA and protein sequences were used for analysis (S1 Table). Phylogenetic trees were generated and we found that eel FPs from the families Anguillidae and Chlopsidae are either sister to the FABP7 brain clade, which diverged from primitive fishes, or nested within it. Although we found non-fluorescent FABPs in *Kaupichthys* and in the transcriptome of *Anguilla*, these FABPs do not contain the Gly-Pro-Pro tri-peptide motif.

Fluorescent proteins from *Anguilla* and *Kaupichthys* arose from a gene duplication event in these fishes, probably in the common ancestor of the two species. The patterns we observed (Fig 7; S1, S2 and S3 Figs) indicate that the larger gene family involved at least three duplication events. Two of these duplications coincide with the 1R and 2R duplications in the common ancestor of vertebrates. The third probably occurred in the common ancestor of eels, and allowed for the neofunctionalization of the duplicated FABP protein into a FP. To examine if the neofunctionalization of the FPs involved sequence specific changes and dN/dS skew, and potentially natural selection, we examined which branches are evolving under different dN/dS skews. The results of this analysis are shown in Fig 8. Two nodes showed statistically significant difference in skew. The node leading to the brain FABP7 in mammals and birds appears to have a dN/dS ratio of > 2.0. The branch leading to the FPs (both *Kaupichthys* and *Anguilla* FPs) has a dN/dS skew >14, indicating strong sequence change in the common ancestor of these eel FPs, such as has been reported for opsins [38] [39]. The fluorescent eel proteins are, therefore, members of a novel family of FABP7 proteins.

A site-by-site analysis of dN/dS skew in the FABP7 family of proteins indicates several sites in the protein that are showing significant skew using the MEME option in Datamonkey [19]. The Gly-Pro-Pro motif was positively selected for during the evolution of FABPs, leading to the evolution of this new fluorescent protein family. It is interesting to note that this result was inferred using different trees, and while not identical, they are overall very similar. Fig 8 maps the location of the inserted Gly-Pro-Pro residues in the middle of the eel FPs. There is also significant change in amino acid function very near to the insertion sites in the FPs. While the skewed sites and changed function sites do not directly coincide with the inserts it is interesting to note that regions adjacent to these do exhibit significant patterns. These results suggest that several cluster sites in the protein are showing significant dN/dS ratio. Of these clusters two are adjacent to residues that are responsible for the fluorescent property of these proteins.

### Expression in Mammalian Cells

**Fig 9:**
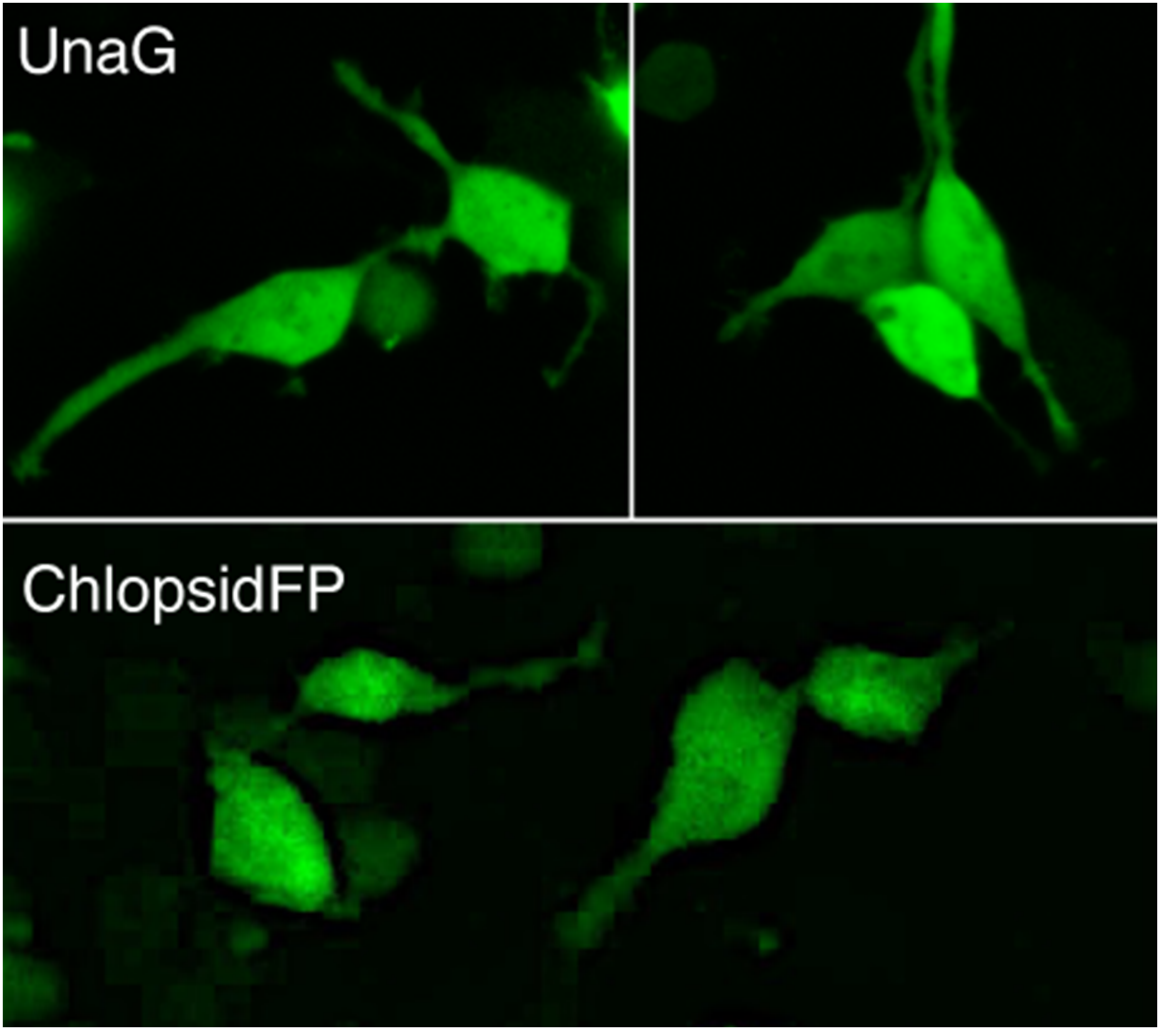
Two photon image of eel fluorescence. UnaG distribution in HEK293 cells (top panel), two photon localization of Chlopsid FP I in HEK293 cells (bottom panel) and confocal images of CiVSP-UnaG fusions in HEK293 cells.

We expressed Chlopsid FP I in mammalian cells (HEK293) without the addition of bilirubin (Fig 9). The cells exhibited bright fluorescence under single and two-photon imaging modalities. The two-photon excitation was fairly flat from 700–1000 nm with a peak at 860 nm and an unusual dip at 840 nm (S4 Fig).

## Discussion

The marine environment has proven to be the richest reservoir of novel FPs on the planet [40]. The upper photic ocean is stably monochromatic with downwelling daylight becoming primarily blue (470-490nm) in color with increasing depth [1]. Over millions of years this stable monochromatic spectral environment likely facilitated the evolution of fluorescent molecules that absorb and re-emit high-energy blue wavelengths into longer, lower energy colors. To date, the two major families of fluorescent molecules with sufficient molar brightness (high cross sections and quantum yield) and expression to produce a fluorescent signal that is visually evident to humans, GFP and the current bilirubin binding proteins discussed here, have evolved exclusively in marine organisms.

The first GFP was discovered in a hydrozoan jellyfish, *Aequorea victoria*, coupled to the bioluminescent apparatus [3], converting blue bioluminescent light to green [41]. GFP orthologs were later found in non-bioluminescent anthozoas [4], primarily scleractinian corals [5]. GFP orthologs have also been discovered in a few additional marine organisms including planktonic copepods [6], lancelets [7], and a ctenophore [9]. However, it was recently discovered that fluorescence is not only phylogenetically widespread, but is also phenotypically variable across both cartilaginous and bony fishes [17].

In the present study, we demonstrate the phylogenetic origins of fluorescent fatty acid binding proteins (FABPs) from marine eels and show the key evolutionary motif switch from FABPs to FPs. FABPs are members of the intracellular lipid binding protein family (iLBP) and are involved in reversibly binding and trafficking a wide range of intracellular hydrophobic ligands. FABPs are small (~16 kDa), structurally conserved cytosolic proteins consisting of a interior binding pocket filled with water, surrounded by ten anti-parallel beta sheets forming a beta barrel [42]. At the surface of the beta barrel, two alpha helices cap the pocket and are thought to be involved in regulating ligand binding [43]. In most vertebrates, there are two major kinds of vertebrate FABPs, those found in the brain and those found in the heart. For the fluorescent FABPs, there is a key Gly-Pro-Pro motif that is essential for fluorescence and is present in all fluorescent FABPs (Fig 8). This family of fluorescent eel FABPs is considerably smaller (~16 kDa) than GFP (26.9 kDa), requires bilirubin for fluorescence, and is oxygen independent.

The order Anguilliformes, the true eels, comprises about 800 species that have traditionally been classified in three major suborders and 111 genera. While *Anguilla* is known to undergo vast migrations (thousands of kilometers) between growth habitats in freshwater and spawning habitats in tropical and subtropical open ocean areas [44], the poorly studied chlopsid eels are not reported to do so. Therefore, the hypothesis that fluorescence acts as a muscle tissue antioxidant mechanism in *Anguilla*, via the non-covalent binding of bilirubin [11], may not hold for *Kaupichthys*. In Chlopsidae there is a possibility that fluorescence serves a visual function. During full moon spawning events, the moonlight could potentially stimulate fluorescence and increase species contrast against the blue background of the ocean for these cryptically patterned, and otherwise reclusive fishes. Little visual or reproductive data are available for *Kaupichthys*, however, calculations suggest that these eels exhibit a lunar cycle of reproduction and that they synchronously spawn during or shortly after full moon periods [45]. It is reported for some *Anguilla* species that as they transition during metamorphosis between an adult freshwater yellow eel and the sexually maturing oceanic silver eel, their visual system changes its spectral sensitivity. The middle-wavelength-sensitive cones shifts from ~550 nm to ~525 nm [46]. Fluorescence in coral has been suggested as a means to increase visual contrast in the monochromatic marine environment. [5][47][48][49] Fluorescence appears to play a role in certain visually guided behaviors in reef fishes [50]. In addition, marine fish fluorescence is especially common and morphologically variable in cryptically patterned lineages [17], providing additional support for the hypothesis that fluorescence serves a visual function for marine organisms. Sexually dimorphic fluorescence patterning has also been observed in some species of marine fishes [17].

However, it should also be noted however that biofluorescence in eels may be merely a secondary effect of the organisms unusual management of bilirubin as eels are known to manage heme metabolites differently than other vertebrates. For example, unlike all other known vertebrates, the blood plasma of *A*. *japonica* is blue-green [51] due a high stable concentration of biliverdin [52]. Once produced, biliverdin is further metabolized into bilirubin. However, altered heme metabolite dynamics are not always associated with the emergence of fluoresncece. For example, lamprey exhibit life cycle dependent bilirubinemia and do not exhibit visible fluorescence, nor do they appear to have a Gly-Pro-Pro containing FABP.

The GFP family has proven to be one of the most useful tools in biomedical science [40]. This current report of the evolutionary consideration of fluorescent fatty acid binding proteins from marine eels that can be autonomously expressed in mammalian cells will expand the toolbox of fluorescent probes available for use in experimental biology. As with GFPs, we find that variations in the primary amino acid sequence of this class of FPs alters the protein’s spectral properties. This finding opens the door to mutagenesis investigations that could produce spectral and structural variants (i.e. circular permutants) in which the fluorescence output can be dynamically varied to produce fluorescent event sensors. These findings also raise questions about the behavioral ecology of the poorly known chlopsid eels and if biofluorescence plays a similar functional role (i.e. communication, predator avoidance, prey attraction) as bioluminescence.

## Supporting Information

S1 Fig Phylogenetic trees generated using MP on DNA and Protein data. See text for details of analysis.
(PDF)

S2 Fig Phylogenetic trees generated using ML on DNA and Protein data. See text for details of analysis.
(PDF)

S3 Fig Phylogenetic trees generated using Baysian on DNA and Protein data. See text for details of analysis.
(PDF)

S4 Fig The two-photon excitation spectra of HEK-293 cells transfected with Chlopsid FP I. (PDF)

S1 Table List FABPs sequences used in this analysis. (XLSX)

S2 Table DNA and protein sequences used in analysis. (TXT)

S3 Table Tree search (MP, ML, Bayes) criteria including the different range of alpha and beta to determine the best tree generated by Datamonkey. (XLSX)

## References

[1] Tyler JE, Smith R.C. (1970) Measurements of Spectral Irradiance Underwater New York: Gordon and Breach Science Publishers.

[2] Beebe W, 1935 Half Mile Down. Lane John, The Bodley Head, London. (1935) Half Mile Down. London: John Lane and the Bodley Head.

[3] Shimomura O, Johnson FH, Saiga Y (1962) Extraction, purification and properties of aequorin, a bioluminescent protein from the luminous hydromedusan, Aequorea. J Cell Comp Physiol 59: 223–239. 13911999 10.1002/jcp.1030590302

[4] Matz MV, Fradkov AF, Labas YA, Savitsky AP, Zaraisky AG, Markelov ML,. (1999) Fluorescent proteins from nonbioluminescent Anthozoa species. Nat Biotechnol 17: 969–973. 10504696 10.1038/13657

[5] Gruber DF, Kao HT, Janoschka S, Tsai J, Pieribone VA (2008) Patterns of fluorescent protein expression in Scleractinian corals. Biol Bull 215: 143–154. 18840775 10.2307/25470695

[6] Shagin DA, Barsova EV, Yanushevich YG, Fradkov AF, Lukyanov KA, Labas YA et al (2004) GFP-like proteins as ubiquitous metazoan superfamily: evolution of functional features and structural complexity. Mol Biol Evol 21: 841–850. 14963095 10.1093/molbev/msh079

[7] Deheyn DD, Kubokawa K, McCarthy JK, Murakami A, Porrachia M, Rouse GW, et al (2007) Endogenous green fluorescent protein (GFP) in amphioxus. Biol Bull 213: 95–100. 17928516 10.2307/25066625

[8] Bomati EK, Manning G, Deheyn DD (2009) Amphioxus encodes the largest known family of green fluorescent proteins, which have diversified into distinct functional classes. BMC Evol Biol 9: 77 10.1186/1471-2148-9-77 19379521 PMC2679011

[9] Haddock SH, Mastroianni N, Christianson LM (2010) A photoactivatable green-fluorescent protein from the phylum Ctenophora. Proc Biol Sci 277: 1155–1160. 10.1098/rspb.2009.1774 20018790 PMC2842807

[10] Hayashi S, Toda Y (2009) A novel fluorescent protein purified from eel muscle. The Japanese Society of Fisheries Science 75: 1461–1469.

[11] Kumagai A, Ando R, Miyatake H, Greimel P, Kobayashi T, Hirabayashi Y, et al (2013) A bilirubin-inducible fluorescent protein from eel muscle. Cell 153: 1602–1611. 10.1016/j.cell.2013.05.038 23768684

[12] Salih A, Larkum A, Cox G, Kuhl M, Hoegh-Guldberg O (2000) Fluorescent pigments in corals are photoprotective. Nature 408: 850–853. 11130722 10.1038/35048564

[13] Bou-Abdallah F, Chasteen ND, Lesser MP (2006) Quenching of superoxide radicals by green fluorescent protein. Biochimica Et Biophysica Acta-General Subjects 1760: 1690–1695. 10.1016/j.bbagen.2006.08.014 PMC1764454 17023114

[14] Field SF, Bulina MY, Kelmanson IV, Bielawski JP, Matz MV (2006) Adaptive evolution of multicolored fluorescent proteins in reef-building corals. Journal of Molecular Evolution 62: 332–U315. 16474984 10.1007/s00239-005-0129-9

[15] Roth MS, Deheyn DD (2013) Effects of cold stress and heat stress on coral fluorescence in reef-building corals. Scientific Reports 3. 10.1038/srep01421 PMC3594756 23478289

[16] Roth MS, Latz MI, Goericke R, Deheyn DD (2010) Green fluorescent protein regulation in the coral Acropora yongei during photoacclimation. Journal of Experimental Biology 213: 3644–3655. 10.1242/jeb.040881 20952612

[17] Sparks JS, Schelly RC, Smith WL, Davis MP, Tchernov D, Pieribone VA, et al (2014) The covert world of fish biofluorescence: a phylogenetically widespread and phenotypically variable phenomenon. PLoS One 9: e83259 10.1371/journal.pone.0083259 24421880 PMC3885428

[18] Heinermann PH (1984) Yellow Intraocular Filters in Fishes. Experimental Biology 43: 127–147. 6398222

[19] Murrell B, Wertheim JO, Moola S, Weighill T, Scheffler K, Kosakovsky Pond SL, et al (2012) Detecting individual sites subject to episodic diversifying selection. PLoS Genet 8: e1002764 10.1371/journal.pgen.1002764 22807683 PMC3395634

[20] Andrews S (2010) FastQC: a quality control tool for high throughput sequence data. Available: http://wwwbioinformaticsbabrahamacuk/projects/fastqc.

[21] Bolger AM, Lohse M, Usadel B (2014) Trimmomatic: a flexible trimmer for Illumina sequence data. Bioinformatics 30: 2114–2120. 10.1093/bioinformatics/btu170 24695404 PMC4103590

[22] Mehr S, Verdes A., DeSalle R., Sparks J., Pieribone V., Gruber D.F. (2015) Transcriptome sequencing and annotation of the polychaete Hermodice carunculata (Annelida, Amphinomidae). BMC Genomics. 10.1186/s12864-015-1565-6 PMC4462082 26059236

[23] Grabherr MG, Haas BJ, Yassour M, Levin JZ, Thompson DA, Amit I, et al (2011) Full-length transcriptome assembly from RNA-Seq data without a reference genome. Nat Biotechnol 29: 644–652. 10.1038/nbt.1883 21572440 PMC3571712

[24] Langmead B, Salzberg SL (2012) Fast gapped-read alignment with Bowtie 2. Nat Methods 9: 357–359. 10.1038/nmeth.1923 22388286 PMC3322381

[25] Pooyaei Mehr SF, DeSalle R, Kao HT, Narechania A, Han Z, Tchernov D, et al (2013) Transcriptome deep-sequencing and clustering of expressed isoforms from Favia corals. BMC Genomics 14: 546 10.1186/1471-2164-14-546 23937070 PMC3751062

[26] Rice P, Longden I, Bleasby A (2000) EMBOSS: the European Molecular Biology Open Software Suite. Trends Genet 16: 276–277. 10827456 10.1016/s0168-9525(00)02024-2

[27] Altschul SF MT, Schaffer AA, Zhang J, Zhang Z, Miller W and Lipman DJ. (1997) Gapped BLAST and PSI-BLAST: a new generation of protein database search programs. Nucleic Acids Research 25: 3389–3402. 9254694 10.1093/nar/25.17.3389 PMC146917

[28] Abascal F, Zardoya R, Telford MJ (2010) TranslatorX: multiple alignment of nucleotide sequences guided by amino acid translations. Nucleic Acids Res 38: W7–13. 10.1093/nar/gkq291 20435676 PMC2896173

[29] Swofford DL (2002) Phylogenetic analysis using parsimony (*and other methods). Version 4, Sunderland, MA: Sinauer and Associates.

[30] Stamatakis A. H P, Rougemont J. (2008) A Rapid Bootstrap Algorithm for the RAxML Web-Servers. Systematic Biology 75: 758–771. 10.1080/10635150802429642 18853362

[31] Posada D (2006) ModelTest Server: a web-based tool for the statistical selection of models of nucleotide substitution online. Nucleic Acids Research 34: W700–W703. 16845102 10.1093/nar/gkl042 PMC1538795

[32] Darriba D, Taboada GL, Doallo R, Posada D (2011) ProtTest 3: fast selection of best-fit models of protein evolution. Bioinformatics 27: 1164–1165. 10.1093/bioinformatics/btr088 21335321 PMC5215816

[33] Atchley WR Z J, Fernandes AD, Drüke T (2005) Solving the protein sequence metric problem Proceeings of the National Academy of Sciences 102: 6395–6400. 10.1073/pnas.0408677102 PMC1088356 15851683

[34] Smith DG B, E B(1989) Family Chlopsidae. Memoirs of the Sears Foundation for Marine Research 1: 72–97.

[35] Meyer A, and Schartl M (1999) Gene and genome duplications in vertebrates: the one-to-four (-to-eight in fish) rule and the evolution of novel gene functions. Current Opinion in Cell Biology 11 6: 699–704 (1999) Gene and genome duplications in vertebrates: the one-to-four (-to-eight in fish) rule and the evolution of novel gene functions.. Current Opinion in Cell Biology 11: 699–704. 10600714 10.1016/s0955-0674(99)00039-3

[36] Dann SG, Allison WT, Levin DB, Taylor JS, Hawryshyn CW (2004) Salmonid opsin sequences undergo positive selection and indicate an alternate evolutionary relationship in Oncorhynchus. Journal of Molecular Evolution 58: 400–412. 15114419 10.1007/s00239-003-2562-y

[37] Porter ML, Cronin DA, Crandall KA (2007) Molecular characterization of crustacean visual pigments and the evolution of pancrustacean opsins. Molecular Biology and Evolution 24. 10.1093/molbev/msl152 17053049

[38] Dann SG, Allison WT, Levin DB, Taylor JS, Hawryshyn C.W. (2004) Salmonid opsin sequences undergo positive selection and indicate an alternate evolutionary relationship in Oncorhynchus. Journal of Molecular Evolution 58: 400–412. 15114419 10.1007/s00239-003-2562-y

[39] Porter ML, Cronin DA, Crandall KA(2007) Molecular characterization of crustacean visual pigments and the evolution of pancrustacean opsins. Mol Biol Evol 24: 253–268. 17053049 10.1093/molbev/msl152

[40] Pieribone V, Gruber DF (2006) Aglow in the Dark: The Revolutionary Science of Biofluorescence Cambridge, MA: Belknap Press of Harvard University Press 288 p.

[41] Morin JG, Hastings JW (1971) Biochemistry of the bioluminescence of colonial hydroids and other coelenterates. J Cell Physiol 77: 305–312. 4397527 10.1002/jcp.1040770304

[42] Storch J, McDermott L (2009) Structural and Functional Analysis of Fatty Acid-Binding Proteins. Journal of Lipid Research 50: 126–131. 19017610 10.1194/jlr.R800084-JLR200 PMC2674722

[43] Smathers RL, Petersen DR (2011) The human fatty acid-binding protein family: evolutionary divergences and functions. Hum Genomics 5: 170–191. 21504868 10.1186/1479-7364-5-3-170 PMC3500171

[44] Tsukamoto K (2006) Oceanic biology: spawning of eels near a seamount. Nature 439: 929 16495988 10.1038/439929a

[45] Lee TW, Miller MJ Hwang HB, Wouthuyzen S, Tsukamoto K (2008) Distribution and early life history of Kaupichthys leptocephali (family of Chlopsidae) in the central Indonesian Seas. Marine Biology 153: 285–295.

[46] Bowmaker JK, Semo M, Hunt DM, Jeffery G (2008) Eel visual pigments revisited: the fate of retinal cones during metamorphosis. Vis Neurosci 25: 249–255. 10.1017/S0952523808080152 18321400

[47] Dove SG, Hoegh-Guldberg O, Ranganathan R (2000) Major colour patterns of reef building corals are due to a family of GFP-like proteins. Coral Reefs 19: 197–204.

[48] Lukyanov KA, Fradkov AF, Gurskaya NG, Matz MV, Labas YA, Savitsky AP (2000) Natural animal coloration can Be determined by a nonfluorescent green fluorescent protein homolog. The Journal of Biological Chemistry 275: 25879–25882. 10852900 10.1074/jbc.C000338200

[49] Mazel CH, Fuchs E (2003) Contribution of fluorescence to the spectral signature and perceived color of corals. Limnology and Oceanography 48: 390–401.

[50] Gerlach T, Sprenger D, Michiels NK (2014) Fairy wrasses perceive and respond to their deep red fluorescent coloration. Proc Biol Sci 281. 10.1098/rspb.2014.0787 PMC4071555 24870049

[51] Kochiyama Y, Yamaguchi K, Hashimoto KN, Matsuura F (1966) Studies on a blue-green serum pigment of eel-I. Isolation and some physico-chemical properties. Bulletin of the Japanese Society for the Science of Fish 32: 867–872.

[52] Fang LS (1984) The identification and occurrence of the chromogen in the blue-green blood of the Japanese eel, Anguilla japonica. Bulletin of the Institute of Zoology, Academia Sinica 23: 1–7.

