# Supplementary figures and images for: Adaptive Evolution of Eel Fluorescent Proteins from Fatty Acid Binding Proteins Produces Bright Fluorescence in the Marine Environment
Source: PLoS One. 2015 Nov 11;10(11):e0140972. doi: 10.1371/journal.pone.0140972 (PMC4641735; doi:10.1371/journal.pone.0140972)

MP

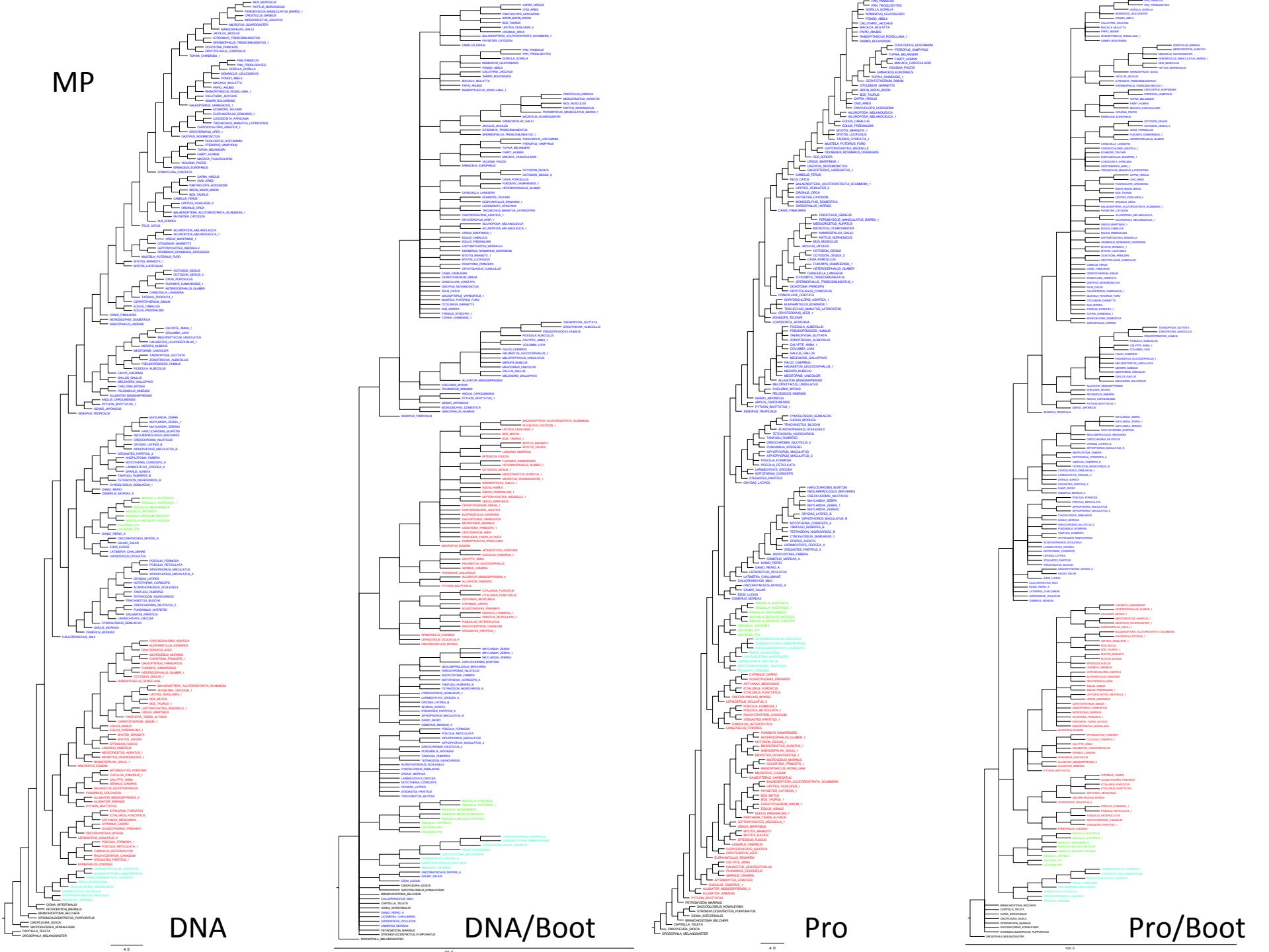

DNA

DNA/Boot

Pro

Pro/Boot

Supplement: S1 Fig — See text for details of analysis. (PDF) [file pone.0140972.s001.pdf]

ML

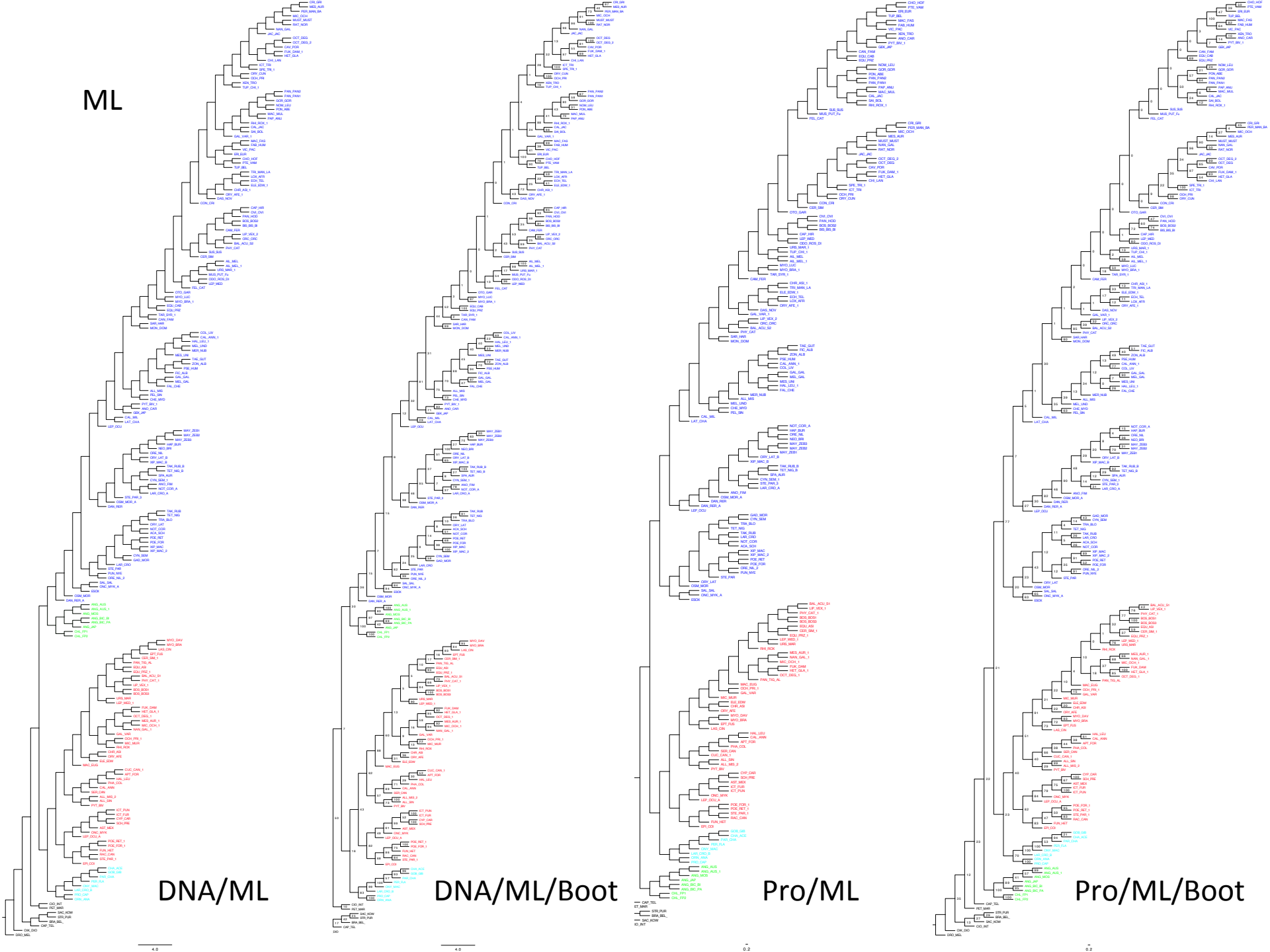

DNA/ML

DNA/ML/Boot

Pro/ML

Pro/ML/Boot

Supplement: S2 Fig — See text for details of analysis. (PDF) [file pone.0140972.s002.pdf]

# Bayesian

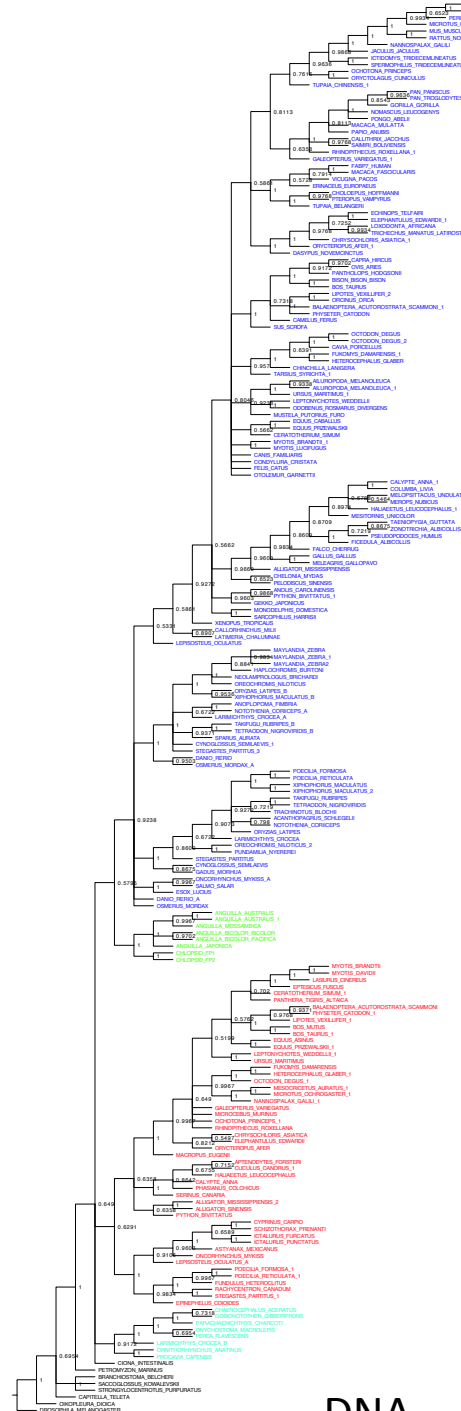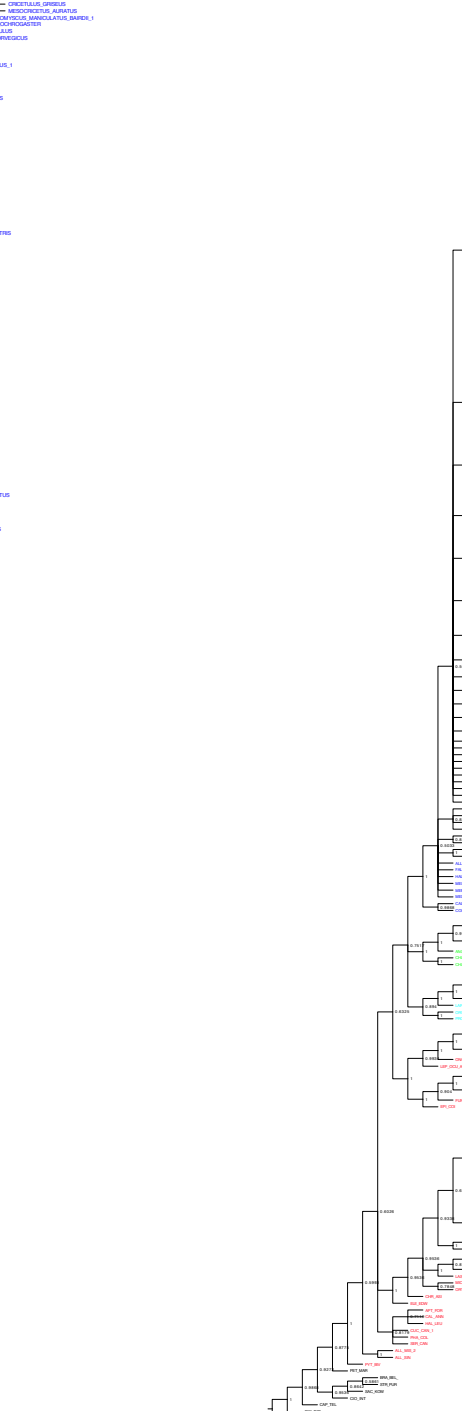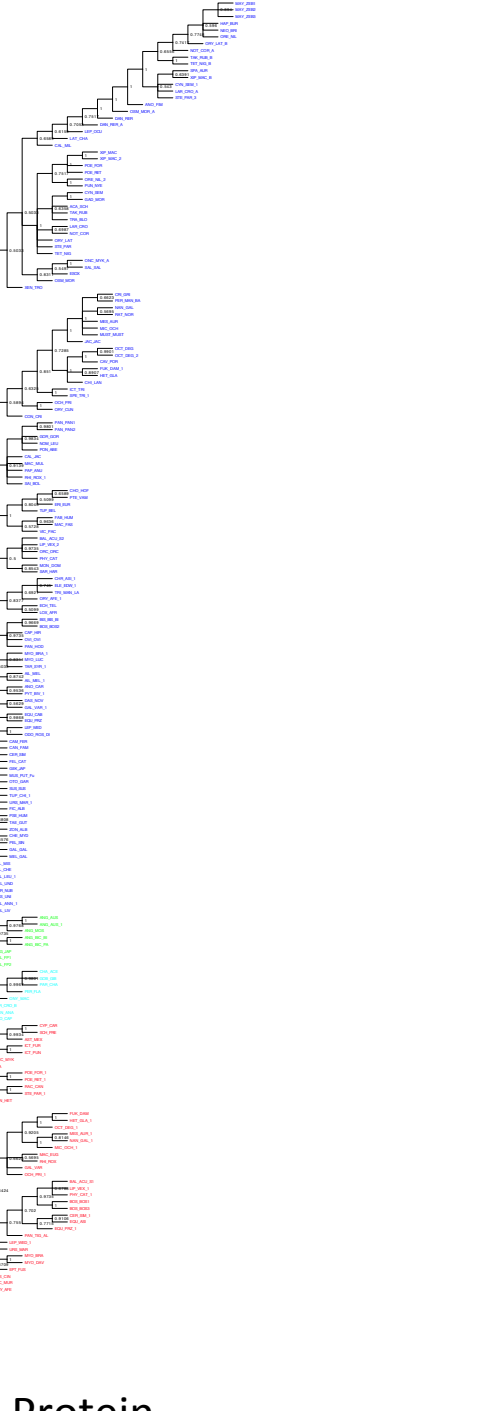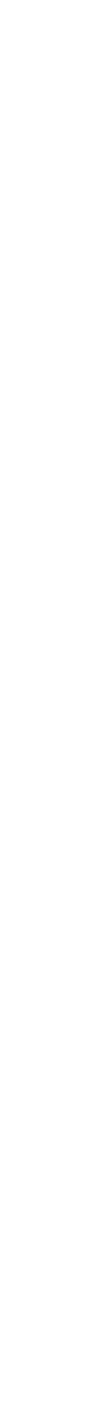

Supplement: S3 Fig — See text for details of analysis. (PDF) [file pone.0140972.s003.pdf]

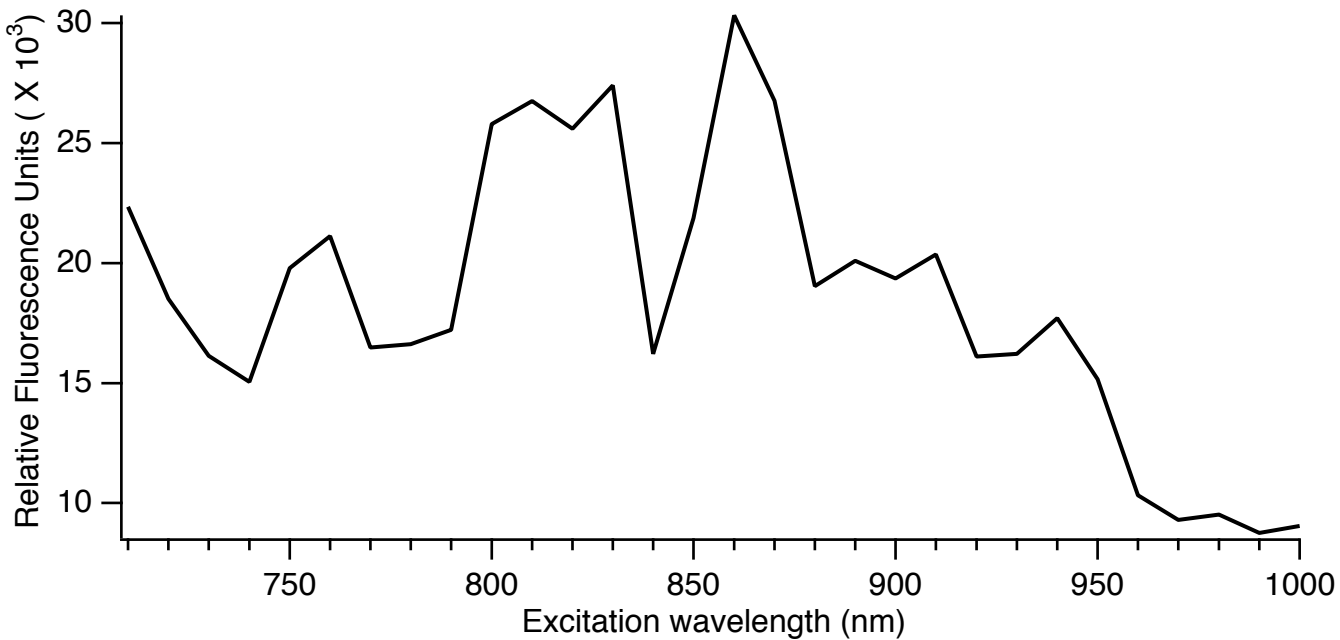

Supplement: S4 Fig — (PDF) [file pone.0140972.s004.pdf]
